# Origin of arc magmatic signature: A temperature-dependent process for trace element (re)-mobilization in subduction zones

**DOI:** 10.1038/s41598-019-43605-9

**Published:** 2019-05-08

**Authors:** Hamed Gamal El Dien, Zheng-Xiang Li, Youngwoo Kil, Tamer Abu-Alam

**Affiliations:** 1 Earth Dynamics Research Group, The Institute for Geoscience Research (TIGeR) and ARC Centre of Excellence for Core to Crust Fluid Systems (CCFS), School of Earth and Planetary Sciences, Curtin University, GPO Box U1987, Perth, WA 6845 Australia; 2 Geology Department, Faculty of Science, Tanta University, 31527 Tanta, Egypt; 3 Department of Energy and Resources Engineering, College of Engineering, Chonnam National University, Yongbong-ro, Buk-gu, Gwangju, South Korea; 4 Universitetsbiblioteket, University of Tromsø - The Arctic University of Norway, 9037 Tromsø, Norway

**Keywords:** Geochemistry, Petrology

## Abstract

Serpentinite is a major carrier of fluid-mobile elements in subduction zones, which influences the geochemical signature of arc magmatism (e.g. high abundances of Li, Ba, Sr, B, As, Mo and Pb). Based on results from Neoproterozoic serpentinites in the Arabian-Nubian Shield, we herein report the role of antigorite in the transportation of fluid-mobile elements (FME) and light rare earth elements (LREE) from the subducted slab to arc-related magma during subduction. The serpentinites contain two generations of antigorites: the older generation is coarse-grained, formed at a temperature range of 165–250 °C and is enriched in Li, Rb, Ba and Cs, whereas the younger generation is finer-grained, formed at higher temperature conditions (425–475 °C) and has high concentrations of B, As, Sb, Mo, Pb, Sr and LREE. Magnesite, on the other hand, remains stable at sub-arc depths beyond the stability field of both antigorites, and represents a potential reservoir of FME and LREE for deeper mantle melts. Magnesite has high FME and LREE absorbing capacity (over 50–60%) higher than serpentine phases. Temperature is the main controlling factor for stability of these minerals and therefore the release of these elements from subducted slabs into arc magmatism. As the liberation of these elements varies along the length of the slab, the resulting cross-arc geochemical variation trend can help to determine the subduction polarity of ancient arcs.

## Introduction

Regardless of the tectonic setting in which they form, serpentinites are a major potential carrier of water (up to 15%) and incompatible fluid-mobile elements (FME) such as Li, B, As, Sb, Pb, Ba, Cs, U and Sr^1–3^ into the subduction zone and the overlying mantle wedge. Serpentinites can be stable to high temperatures (620 °C) and pressures (5 GPa) at depths of up to 150–200 km^4^, and their hydration and subsequent dehydration critically influence the generation and chemistry of arc-related magmas^5–7^. Thus, understanding the role of serpentinites (i.e., dehydration/devolatilization) in trace element geochemical cycles in subduction zones can discriminate between arc-related (i.e., high abundances of FME), plume-related, and mid-ocean ridge (MOR) basalts, and can track the cross-arc geochemical variations^8^. Currently, the factors and mechanisms that control the transfer of these elements from oceanic environments through subducting serpentinites to arc magmatism are still unclear.

Numerous *in situ* studies were previously carried out on serpentinites with the aim of defining the behavior of fluid-mobile elements and rare earth elements (REE) during subduction. These studies highlighted the role of the original minerals (i.e., olivine vs. pyroxene) and temperature on trace element distributions during the subduction process^5,9–12^ but did not consider the distribution of trace elements at higher-temperature conditions within the stability field of antigorite. Instead, they concentrated on the lizardite/antigorite transition. In addition, they did not provide enough constrains on the temperatures range beyond which FME and REE are uptaken/released from the serpentines to arc magma. Although subducted carbonates are recognized to be a potential source of C for deep mantle melts through subduction^13,14^, the role of carbonate minerals in serpentinite-bearing rocks as a reservoir for FME and REE has not been adequately considered.

Here, we use the results of a detailed petrological, mineralogical and geochemical study from selected Neoproterozoic serpentinite bodies in the Arabian-Nubian Shield (a typical arc-accretion orogen)^15^ to constrain the role of temperature within the stability field of antigorite on the distribution of FME and REE in subduction zones. In addition to antigorite, mineral chemistry of carbonate minerals indicates that magnesite is a potential reservoir for these elements during the subduction process. Our work argues against the prevailing view that original minerals (olivine and pyroxene) are the main factors controlling the trace element distribution. Instead, we provide evidence for the systematic distribution of FME and REE in subduction zones that depends on temperature conditions of the subducting slab, and we illustrate how the resultant cross-arc geochemical trends may help to determine the subduction polarity of ancient arcs in Earth’s history.

### Geological background and sample description

The Arabian-Nubian Shield (ANS) represents the largest Neoproterozoic juvenile continental crust. ANS formed through accretion of island arcs to the Gondwanan continental margins by the closure of the Mozambique Ocean during the East-African orogeny (750–550 Ma)^16^. ANS ophiolites (which include abundant serpentinites) mark the suture zones between the accreted arcs^15^. Egyptian ophiolites, including ultramafic bodies, occur mainly in the central and southern parts of the Eastern Desert, extending across the border with Sudan (Supplementary Fig. S1a). They were intensely deformed in the late Neoproterozoic during oblique collision^17^ and accretion of island arcs onto the Saharan Metacraton, forming ophiolitic mélanges^15^. Although, some ophiolites were previously thought to have formed in mid-ocean ridges^18^, there is a general consensus that the majority of the ANS ophiolites formed in subduction-related tectonic settings^15,19–25^.

Serpentinite samples were collected from Wadi Muweilih in the Central Eastern Desert of Egypt (Supplementary Fig. S1b,c). This area is part of the Um Esh-Um Seleimat tectonic ophiolitic mélange^26^ (Supplementary Fig. S1b), and consists of highly sheared mélange rocks containing many isolated masses of serpentinites, metagabbros, pillowed metabasalts, schistose rocks, metasedimentary rocks including metaconglomerates^27^ (Supplementary Figs S1c, S2a,b). These serpentinized bodies crop out either as large lenticular blocks about1–3 km in size, or as small thrust sheets in a tectonic mélange with small amounts of talc-carbonate rocks. The contacts of the serpentinites with metasedimentary and metagabbroic rocks in the east and with metaconglomerates in the west are both SW-dipping thrusts (Fig. S1c). Along the faults, sheared serpentinites have been altered to talc-carbonates.

**Figure 1:**
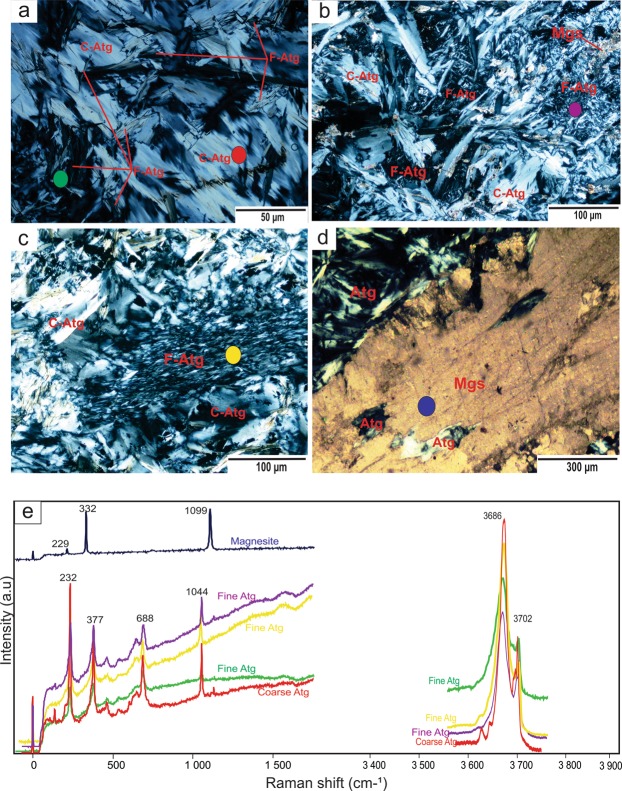
Photomicrographs of the studied serpentinites. (**a–c**) Interpenetrating texture of antigorite (Atg). (**d**) Magnesite (Mgs) clast containing antigorite relics. (**e**) Raman spectra of fine (F) and coarse (C)-grained antigorites in the studied serpentinites in low frequency (0–1500 cm^−1^) and OH stretching (3350–3900 cm^−1^) ranges. Antigorite shows discrete peaks at 232, 377, 688 and 1044 cm^−1^ in the low wavenumber region and 3686 and 3702 cm^−1^ in the OH-band region. The Raman spectra of magnesite show peaks at 229, 332 and 1099 cm^−1^ in the low wavenumber region.

Petrographic investigations and Raman spectral analyses reveal that the protolith of the studied peridotites was suffered variable degrees of serpentinization and carbonation. Primary minerals olivine and pyroxene are completely serpentinized and chrome-spinel is the only primary relict phase. The studied serpentinites are composed mainly of antigorite, magnesite ± dolomite, magnetite, small amounts of chrome-spinel, and minor amounts of chlorite and talc (Supplementary Fig. S2d,e). Antigorite exhibits a predominantly a non-pseudomorphic, interpenetrating texture (Fig. 1a–c) and mainly occurs in two distinct crystal sizes: fine aggregate grains (20–50 µm) and coarse radiating fibrous (100–150 µm) (Fig. 1a–c). The fine antigorites commonly overprint the coarse ones (Fig. 1a–c). Magnesite is present in two forms: either as anhedral clusters (Fig. 1d) or in small veinlets associated with fine dolomite (Supplementary Fig. S2c,d). Magnesite, magnetite, chrome-spinel, chlorite and talc occur as randomly distributed grains among antigorite-dominated groundmass.

## Results

### Geochemical composition

**Figure 2:**
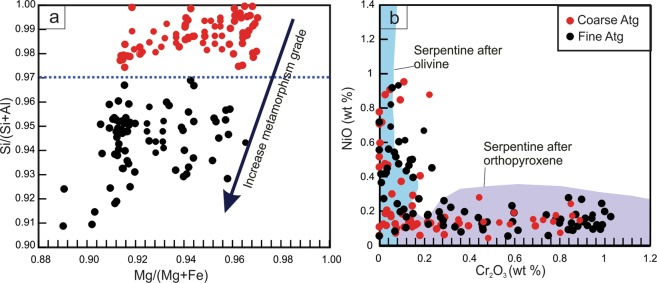
Mineral chemistry of antigorites from the studied samples. (**a**) Si/ (Si + Al) vs. Mg/ (Mg + Fe). The variation of Mg/ (Mg + Fe) indicates different bulk chemistry of the rocks while the variation of Si/ (Si + Al) is due to increase in the metamorphic grade. (**b**) NiO vs. Cr_2_O_3_ (wt %). Data used to create olivine and orthopyroxene fields collected from Kodolányi *et al*.^12^.

**Figure 3:**
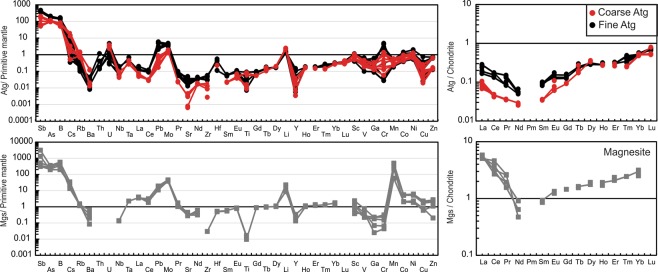
Primitive mantle^29^ normalized multielement and rare earth element patterns normalized to chondrite^28^ of the studied antigorite and magnesite.

All the studied serpentinite samples have high-water contents (i.e., high LOI values), ranging from 12.54 to 14.93 wt% (for details on the bulk-rock compositions see Supplementary Information). The Raman spectra of the examined serpentinite samples identify antigorite as the only serpentine phase (Fig. 1e). Two groupings of antigorite are distinguished based on their different textures (grain sizes) and chemical compositions (Figs. 1 and 2a; Supplementary Table). The first group is coarse-grained (100–150 µm), and the second group is fine-grained (20–50 µm) (Fig. 1a–c). The coarse-antigorite (CA) group has higher SiO_2_ content (43.01–45.86 wt%) and lower Al_2_O_3_ content (0.02–0.97 wt%) compared to the fine-antigorite (FA) group which range in SiO_2_ from 39.52–42.95 wt% and in Al_2_O_3_ from1.16–3.38 wt% (Fig. 2a). MgO and FeO contents do not show systematic variation between the two groups (Fig. 2a). NiO (up to 0.92 wt %) and Cr_2_O_3_ (up to 1.06 wt %) contents show no notable difference, and both groups plot in the serpentine fields of olivine and orthopyroxene, reflecting a harzburgite protolith (Fig. 2b). *In situ* trace element analyses reveal a heterogeneous distribution of FME between the CA- and FA-groups. FME such as B have higher contents in the FA-group (40.89–49.87 ppm) than in the CA–group (15.32–24.50 ppm), whereas Li has higher contents in the CA-group (3.44–3.95 ppm) than in the FA-group (2.03–2.73 ppm) (Fig. 3; Supplementary Fig. S6, Table). Moreover, the CA-group has lower contents of As, Sb, Pb, Mo, Sr and higher contents of Rb, Cs and Ba (Fig. 3; Supplementary Fig. S6, Table). Th, U, P and high-field strength elements (HFSE: Nb, Ta, Zr and Hf) show no obvious difference between the two groups, but Ta and Hf show positive spikes compared to elements of the same compatibility in both groups (Fig. 3). REE normalized to (CI)-Chondrite^28^ patterns have a U-shape pattern, and the light rare earth elements (LREE) contents higher in the FA-group (La = 0.074–0.127 ppm, La_N_/Sm_N_ = 1.96–3.29) compared to the CA-group (La = 0.031–0.047 ppm, La_N_/Sm_N_ = 1.88–2.79) (Fig. 3). The middle rare earth elements (MREE) to heavy rare earth elements (HREE) show a slight negative slope. The HREE concentrations of the FA and CA- groups are indistinguishable (Yb_N(CI-normalized value)_ = 0.47–0.57) (Fig. 3).

Magnesite is the main carbonate mineral, representing 5–10% of the modal mineral composition in the studied samples, although minor thin veinlets of dolomite also occur (Fig. 1d; supplementary Fig. S2c,d). Magnesite composition varies in MgO (37.72–40.49 wt %), FeO (1.88–5.77 wt %) and MnO (0.32–2.74 wt %). Dolomite has variable contents of MgO (14.83–20.21 wt %), CaO (21.44–27.54 wt %), FeO (0.44–5.65 wt %) and MnO (0.09–1.15 wt %). A Primitive Mantle (PM)-normalized^29^ trace element diagram of analysed magnesite shows a significant enrichment in incompatible elements (Fig. 3). Almost all FME, such as Sb (1.55–17.83 ppm), B (59.06–170.96 ppm), As (25.31–46.30 ppm), Mo (1.89–2.28 ppm), Cs (0.28–0.72 ppm), Li (13.90–36.01 ppm) and Pb (1.63–2.86 ppm) are enriched in magnesite (Fig. 3; Supplementary Table). Rb (0.70–0.91 ppm) is slightly enriched and both Sr (5.48–9.04 ppm) and Ba (0.56–4.32 ppm) are slightly depleted relative to primitive mantle but are slightly enriched relative to elements with similar compatibility (Fig. 3). HFSE such as Ti (11.62–20.39 ppm), Zr (~0.31 ppm), Hf (0.14–0.15 ppm), Nb (0.08–0.09 ppm) and Y (0.57–1.44 ppm) are highly depleted. Chondrite^28^-normalized REE pattern shows the same U-shape like antigorites, but with higher LREE contents (La_N_ = 5.04–5.80) (Fig. 3). HREE are highly enriched, with Yb up to 3 times of that of Chondrite, and show a slight negative slope to MREE.

### Thermodynamic modelling

**Figure 4:**
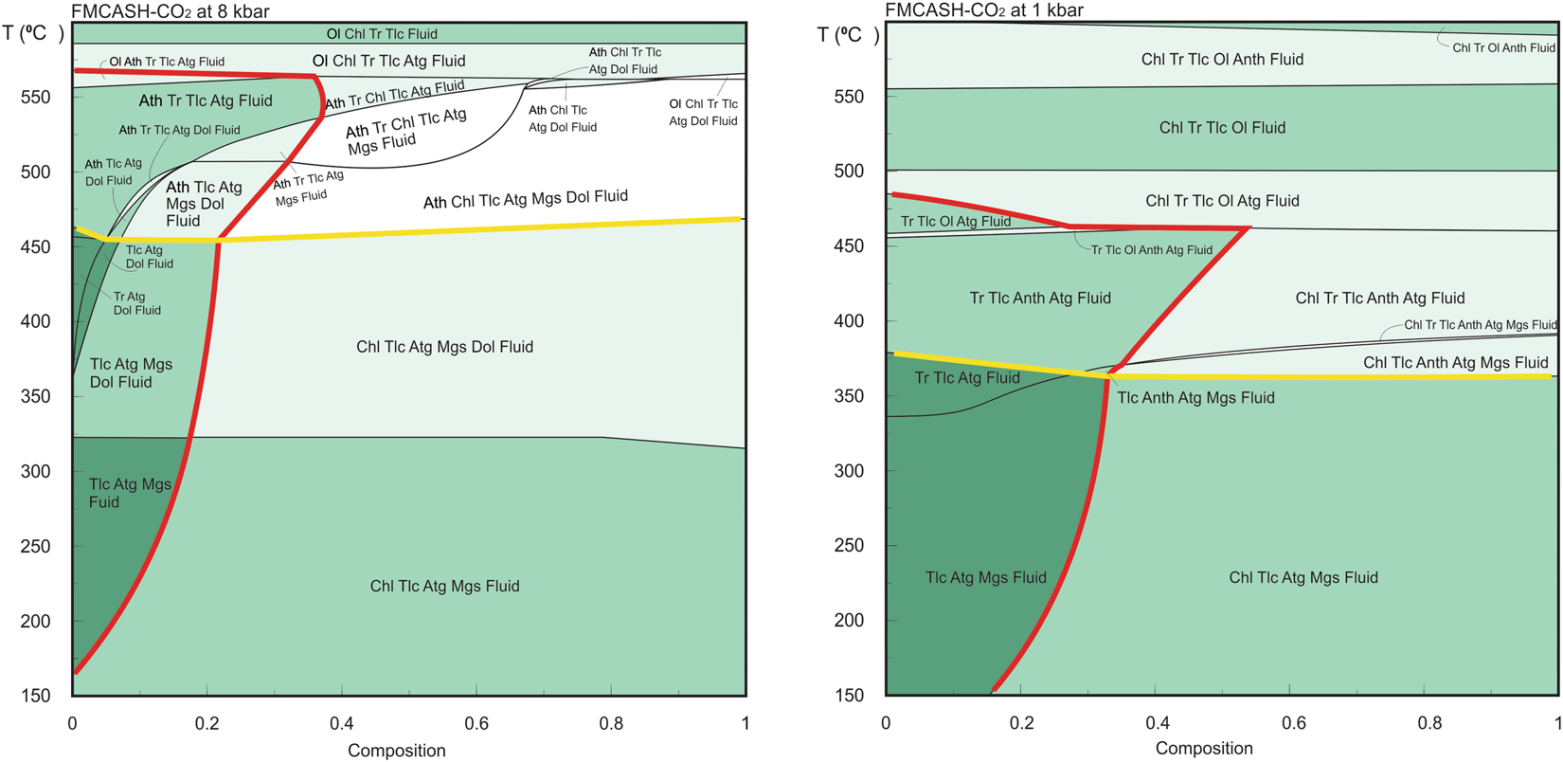
T-X pseudosections. The two pseudosections constructed at pressure 8 kbar and 1 kbar in the chemical system FMCASH-CO_2_ using the activity models cited in the methods section. The x-axes represent a range of the bulk composition in wt% where 0 is equivalent to bulk of: FeO: 4.157, MgO: 47.13, CaO: 0.001, Al_2_O_3_: 0.715, SiO_2_: 40.524, H_2_O: 8.89, CO_2_: 0.001, and 1 is equivalent to bulk of: FeO: 7.00, MgO: 35.00, CaO: 0.04, Al_2_O_3_: 3.00, SiO_2_: 50.00, H_2_O: 18.00, CO_2_: 0.8. The red isochemical lines represent the chlorite-in boundaries. The studied mineral assemblages are stable to right-side of the chlorite-in boundaries. The yellow line shows the anthophyllite-in boundary. The studied assemblages are stable to the lower temperature part of the anthophyllite-in boundaries.

**Figure 5:**
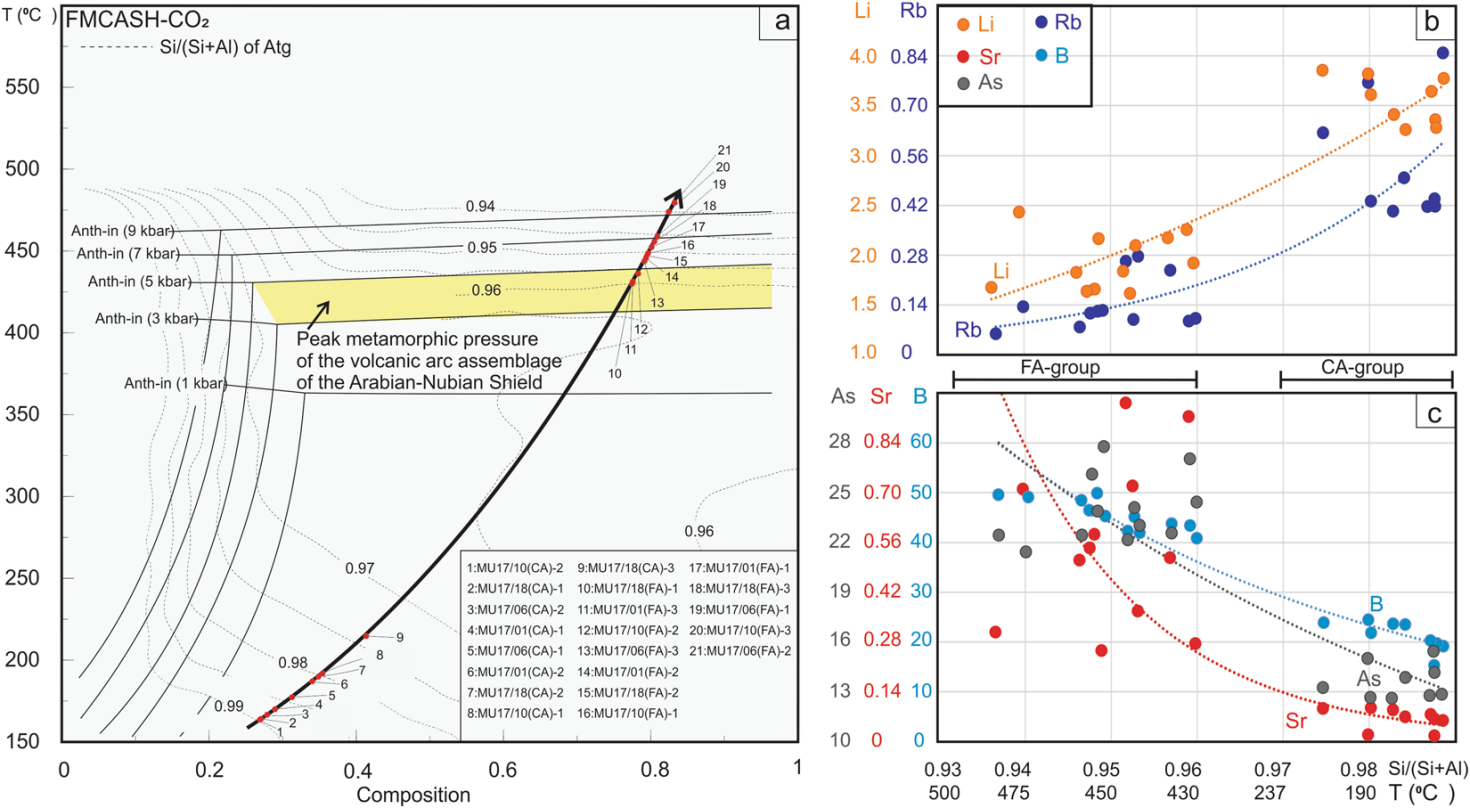
P-T-X pseudosection showing the shift of the anthophyllite-in boundaries toward higher temperature conditions when the pressure increases (**a**). The yellow area represents the anthophyllite-in boundaries at pressure of ca. 4 kbar which is equivalent to the peak metamorphic pressure of the volcanic arc assemblages of the Arabian-Nubian Shield^83^. The pressure represents a combination of lithostatic and hydrostatic pressures. The vertical isochemical solid lines show the shifting in the chlorite-in boundary with pressure change. Contour lines are the Si/ (Si + Al) ration of the antigorite chemistry in the T-X space. The solid black arrow represents the P-T-X path of the studied rocks. The red dots along the P-T-X path represent some of the analysed antigorites (CA at the low temperature conditions) and (FA at the high temperature conditions). The right hand plots shows the variations of Li and Rb (**b**) B, Sr and As (**c**) contents of the two group antigorites with the temperature changing along the P-T-X path. The x-axes represents temperature and Si/(Si + Al).

A series of T-X pseudosections at different pressures were calculated to estimate the pressure-temperature conditions of the serpentinization. All the T-X pseudosections show stability of the spinel mineral at high-temperatures (>670 °C; Supplementary Fig. S7). These temperature estimates indicate that the observed spinel in the studied samples is a metastable phase with respect to the surrounded low-grade assemblages^30^. Figure 4 shows two T-X pseudosections at 1 and 8 kbar, respectively. These pseudosections have similar topology but dolomite is unstable at low pressure conditions (i.e., 1 kbar) within the studied T-X range. At higher pressure conditions (i.e., 8 kbar; Fig. 4), however, dolomite is stable within the temperature range of 325–550 °C. The observed mineral assemblages, i.e., chlorite-talc-antigorite-magnesite-fluid ± dolomite, are thermodynamically stable at the bulk composition range of between 0.15–1 and 0–1 for pressures of 1 and 8 kbar, respectively (Fig. 4). The upper temperature limits for the stability of the observed assemblages are marked by the anthophyllite-in boundaries. At low pressure conditions (e.g. 1 kbar), the anthophyllite-in boundaries appear in the temperature range of 360–375 °C, but at 8 kbar the boundaries shift to the higher temperature (450–475 °C). Figure 5a shows the shift of anthophyllite-in boundaries toward higher temperature with an increase in pressure from 1 kbar to 9 kbar.

The antigorites display an increase in SiO_2_ content and decrease in Al_2_O_3_ content from the FA-group to the CA-group (Fig. 2a; Supplementary Table). Tschermak’s substitution in antigorites is proposed to occur following the equation of M^2+^ + Si^4+^  = Al^3+^ + M^3+^, where M^2+^ resides in the octahedral site (Mg, Fe^2+^, Mn and Ni) and M^3+^ in the tetrahedral site (Fe^3+^, Cr, Al)^31^. Thus, Al^3+^ is incorporated in antigorite through a coupled exchange when one Mg and one Si cation are substituted by two Al cations^31,32^. The Si/(Al + Si) ratio of antigorite has been used as a non-linear potential geothermometer for serpentinite rocks, due to its sensitivity to temperature^32,33^. In the current study, the Si/(Al + Si) ratios are insensitive to changes in pressure but are sensitive to changes in temperature (Fig. 5).

Although the difference in Si/(Al + Si) between the two groups is relatively small (Fig. 5b,c), a gap in the mineralogical composition is clearly observed at Si/(Al + Si) between 0.965 and 0.975 (Figs. 2a and 5b,c) which corresponds with the petrographic transition from the CA to the FA-group. Also, this gap corresponds with the isopleth line (with value 0.965) which is observed in the temperature range of 250–425 °C (Fig. 5a). The CA-group has Si/(Al + Si) in the range of 0.975–1 (apfu; atoms per formula unit), corresponding with a temperature range of ca. 200 to 250 °C (Fig. 5). The FA-group has a Si/(Al + Si) content range of 0.92–0.96 which indicates higher temperature conditions (425–475 °C). Such temperatures cannot be achieved unless the system attains local equilibrium at pressure >9 kbar.

## Discussion

### Origin of the protolith

Field, petrographical and geochemical characteristics of the studied serpentinites suggest a subduction channel origin for the protolith. A subduction channel typically consists of fragments of subducted oceanic serpentinites, metasediments, and altered mafic crust that can be later accreted to the arc system to form at mélange zone^34–36^. Such serpentinites are typically strongly sheared and dominated by antigorite^37^. All of these features are present in the studied region (Supplementary Figs. S1, S2a,b) and most of the ANS ophiolites^15^. The studied serpentinites occur in one of the well-developed suture zones in the Eastern Desert of Egypt, i.e., the Um Esh-Um Seleimat tectonic mélange^26^ (Supplementary Fig. S1b,c). Furthermore, these rocks are similar to some subducted serpentinites in Mesozoic ophiolites such as those of the Zagros suture zone in Iraq^38^ and ophiolite complexes of Northwest Anatolia in Turkey^39^. Previous work (e.g.^20,24^) on ophiolites in the vicinity also suggest a subduction-related origin.

The harzburgite protolith of the studied serpentinites suggests that the peridotite parents have a refractory origin, and clinopyroxenes were almost completely exhausted by pre-serpentinization partial melting (for details on the nature of the protolith see Supplementary information). Moreover, mineralogical (spinel Cr# >0.6)^30^ and geochemical characteristics such as low Al_2_O_3_/SiO_2_ (≤0.04), strong depletion in compatible trace elements (i.e., HREE and Y), over-enrichment of As, Pb, Mo and nearly flat REE patterns, also point to a subducting-slab origin^2,11,34^ for the studied serpentinites and distinguishes them from mantle wedge/fore-arc serpentinites (e.g., Izu-Bonin-Mariana)^12,40^ (Supplementary Figs. S4, S5).

The high field strength element (HFSE: Nb, Th, Hf and Ti) contents of the studied rocks (Supplementary Fig. S5b) differ significantly from patterns expected of a melt residue^41^. In addition, the absence of any correlation between these elements and LOI indicates that the enrichment processes were not related to the serpentinization; instead, they were more likely caused by melt-rock interaction^12,41^. The high Ti content (150–360 ppm) of the studied serpentinites that plot above the melting trend (Supplementary Fig. S5b) suggests interaction of the subducting serpentinite mainly with Ti-rich melts (Ti = 30–500 ppm)^2^. On the other hand, the positive correlations of LREE and FME with LOI point to the enrichment of these elements during serpentinization process of the protolith through fluid/rock interactions (Supplementary Fig. S3).

### Two-stage serpentinization and geochemical consequences

We suggest that the studied rocks experienced two stages of serpentinization which correspond to the different temperature ranges associated with the formation of CA- and FA-group antigorites (Fig. 5). The first stage resulted in the formation of coarse antigorites at 200–250 °C that may have grown directly from the original olivine and orthopyroxene^42^ during the initial stage of subduction. During this stage, the slab-derived fluids caused strong hydration of the subducted peridotites protolith forming CA-group, such as the antigorite serpentinites (≥200 °C)^43,44^ in the Happo-O’ne area in Japan^45^ and some of the other antigorite serpentinites in Eastern Desert, Egypt^21,23^. Although the coarse antigorites could also have crystallized by progressive replacement of lizardite formed during low-temperature oceanic serpentinization^43,46^, we favour the former explanation due to the absences of lizardite relics (i.e., mesh cells)^37^ in the studied serpentinites. The second serpentinization stage occurred at increased subduction depths with elevated metamorphic temperatures of 425–475 °C^10,11^ (Fig. 5), resulting in the formation of the fine antigorites which overprinted and replaced some of the coarse ones (Fig. 1a–c).

**Figure 6:**
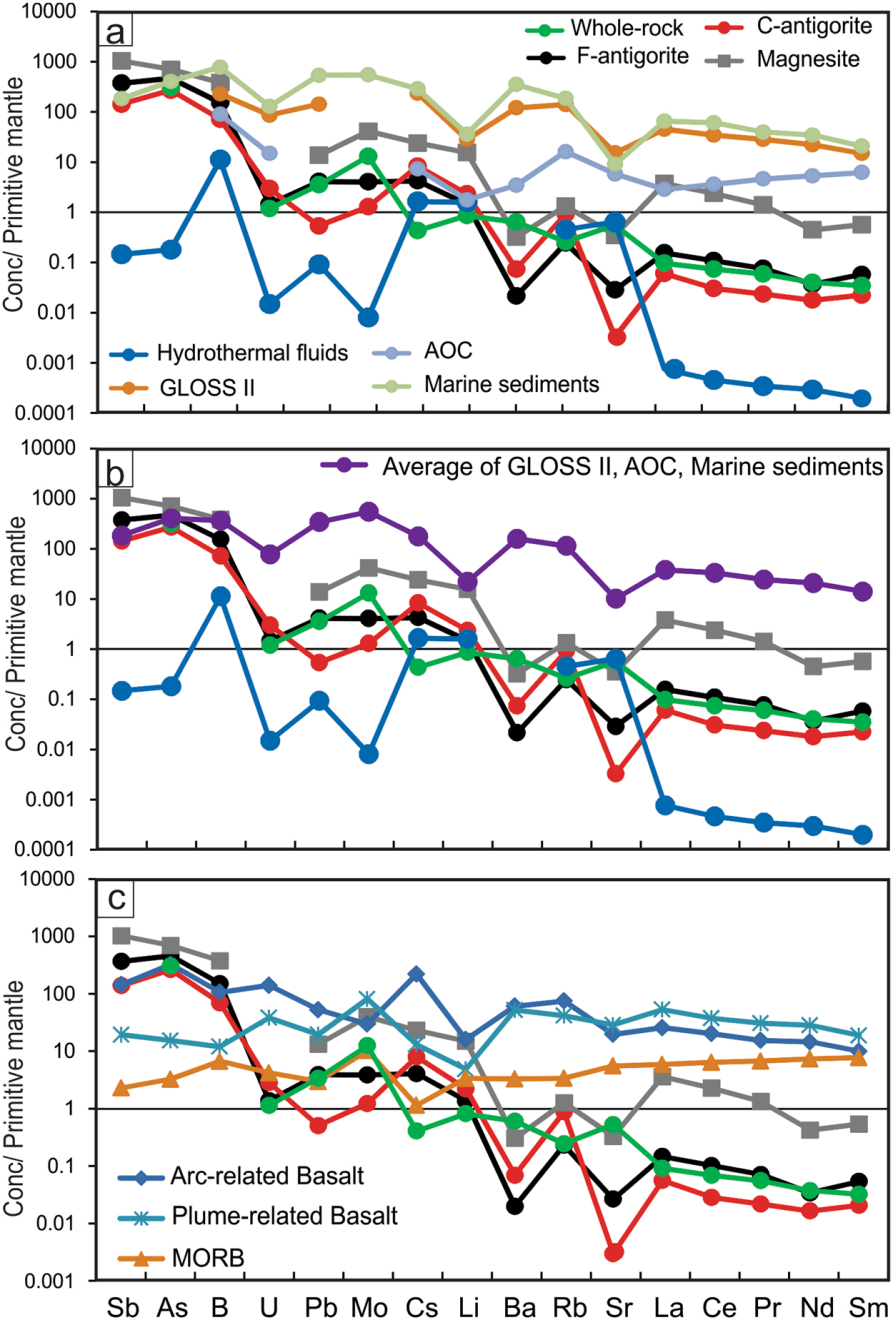
The average contents of fluid-mobile element and light rare earth element patterns of the studied serpentinites and rock forming minerals (antigorite and magnesite) normalized to the Primitive Mantle^29^. (**a,b**) plotted against the composition of hydrothermal fluids (Logatchev, Rainbow, Snake Pit)^54^ and the contents of subduction inputs including altered oceanic crust (AOC)^51^, global subduction sediments (GLOSS II)^52^ and marine sediments pattern^53^ are reported for comparison; (**c**) plotted against the average compositions of arc-related basalts, plume-related basalts and mid ocean ridge basalts (MORB) after Georoc repository (http://georoc.mpch-mainz.gwdg.de/georoc/).

During subduction, oceanic lithosphere gets serpentinized through interactions with circulating fluids released from the subduction channel^3,36,37,47–49^. Although some studies show that the oceanic lithosphere can be serpentinized before it enters the subduction zone^50^, the primitive mantle-normalized pattern of the FME and LREE of the studied serpentinites (Fig. 6) and their rock-forming minerals (antigorites and magnesites) have significant similarity to the subduction inputs components that include mainly altered oceanic crust (AOC)^51^, global subducted sediments (GLOSS II)^52^ and marine sediments^53^. These elements, with the exception of Sr, Rb and Li, are more highly enriched than seawater-derived hydrothermal fluids in spreading mid-ocean ridges (MOR: Logatchev, Rainbow, Snake Pit)^54^. Moreover, the over-enrichments of As, Sb, B and Mo in the studied serpentinites and their rock-forming minerals support an important role for sediment-derived fluids^48,55,56^ which are characteristic of subduction-related serpentinites^2,11,34^. The similarities in Li, Sr and Rb contents between the serpentinites and seawater-derived hydrothermal fluids suggest contribution of seawater either through direct infiltration into fractures and faults that formed during the bending of a subducted slab^57^ as it entered the subduction channel, or through the circulation of water/fluids in the subduction channel that were released from the subducted oceanic lithosphere and marine sediments^58^.

To further test our interpretation, we calculated the FME composition of the fluids that interacted with the studied rocks during serpentinization. There are only a few experimental studies focused on FME (i.e., B, Li, As, Sb and Cs) partitioning during progressive serpentinization^59,60^ and those studies are limited to the low temperature serpentine phase (200–300 °C). Here we used the partitioning coefficient of those elements^59,60^ to estimate the fluid composition in equilibrium with the CA-group antigorites. The estimated composition of those elements in the equilibrated fluids have B = 26.41–42.24 ppm, Li = 0.40–0.46 ppm, Sb = 0.03–0.09 ppm, As = 3.26–3.96 ppm and Cs = 0.07–0.23 ppm. With the exception of Li, these estimates are similar to the subduction input components compositions of GLOSS II and AOC (e.g. B = 26.6–67.9 ppm and Cs = 0.15–4.9 ppm)^51,52^, but are higher than seawater-derived hydrothermal fluids in MOR (B = 3.35 ppm, Cs = 0.03 ppm, As = 0.009 ppm and Sb = 0.0008 ppm)^54,61^. Therefore, we conclude that the studied serpentinites were formed in a subduction-related environment where serpentinization was caused mainly by fluids from the subduction channel with sedimentary input.

The studies of Bebout^48^ and Marschall *et al*.^62^ demonstrated the release of B, Li, As, Sb and Cs from sedimentary and mafic rocks via prograde metamorphism and increasing pressure-temperature conditions within the subduction channel. *In situ* B and Li analyses in the studied antigorites indicate, based on thermodynamic modelling (Figs. 4, 5), that the different distributions in B and Li between the two antigorite groups (CA- av. = 21.29, 3.69 ppm, respectively; FA- av. = 45.61, 2.30 ppm, respectively) (Figs. 5b,c and 6) are related to different temperature conditions. The enrichment of B at high temperature and Li at low temperature contrasts with some previous studies^2,12,34,63^ which argue that B enrichment occurs in the low temperature serpentine phase and is depleted at high temperature conditions. However, it is in agreement with the reported retention of B at higher metamorphic grade phase^5,64^. Our interpretation is also supported by a continuous loss of B from the subducting slab (i.e., low B at high temperature) during the progression of subduction^48^. The behaviour of Li, on the other hand, is in agreement with previous studies that revealed the loss of Li during the lizardite/antigorite transition and increasing temperature of serpentinization. Furthermore, As, Sb, Pb and Mo contents are higher in the high temperature FA-group (Av. As = 23.16 ppm, Sb = 2.04 ppm, Pb = 0.61 ppm, Mo = 0.20 ppm) compared to the low temperature CA-group (Av. As = 13.36 ppm, Sb = 0.77 ppm, Pb = 0.08 ppm, Mo = 0.06 ppm) (Figs. 5c and 6), suggesting addition/incorporation of those elements to the FA-group at higher temperatures. These trends are consistent with a continuous release of those elements from subducted sediments and from AOC during prograde metamorphism^48,56^. Whereas B, As and Sb are preferentially incorporated into tetrahedral Si in sheet silicates^5,6^ (i.e., antigorite), they are likely to increase from CA- to FA-group with decreasing Si/(Al + Si) and increasing temperature (Fig. 5b,c).

Contrary to the aforementioned elements, large ion lithophile elements (LILE) such as Rb, Ba, Cs and U are more enriched in the low temperature CA-group (Av. Rb = 0.55 ppm, Ba = 0.48 ppm, Cs = 0.17 ppm, U = 0.06 ppm) than in the high temperature FA-group (Av. Rb = 0.15 ppm, Ba = 0.14 ppm, Cs = 0.08 ppm, U = 0.02 ppm), consistent with the enrichment of these elements at low-temperature conditions^2^ (Figs. 5b, 6). This enrichment suggests the release of these elements during the high-temperature serpentinization process^11,12,34^. However, Sr has higher content in the high temperature FA-group (Av. = 0.56 ppm compared to Av = 0.06) indicating continuously addition of Sr to the antigorite from the subduction channel-derived fluids and retention at higher temperatures. This result is contrary to previous studies that argue against Sr enrichment at high temperature^2,11,12,34,65^, although those studies concentrated only on lizardite/antigorite transition without determining the formation temperature for each phase or distinguishing between the two temperature-dependant phases of antigorite. Moreover, our results support Kodolányi *et al*.’s^12^ observation that the distribution of B and Sr is controlled by the same mechanisms, which we suggest to be temperature- and fluid-dependent processes.

The REE contents of the serpentine phases are commonly assumed to be an inherited feature from the original minerals (olivine and pyroxene)^2,9,11,12^. Here, the studied antigorite (both the CA- and FA- groups) displays interpenetrating textures with no preservation of the primary minerals, arguing against the role of the parent minerals in the trace element concentrations of antigorite. Although, the two groups have similar HREE contents, the high temperature FA-group have higher LREE (La = 0.074–0.127 ppm) than the low temperature CA-group (La = 0.030–0.047 ppm) (Fig. 6). Our data suggest re-mobilization of LREE with increasing serpentinization temperature.

### Carbonates formation and trace element budget

The predominance of magnesite in the studied serpentinites also indicates a paleo-subduction zone origin^66^ as magnesite is rarely found in carbonate-related ultramafic rocks in normal oceanic settings^66^. Magnesite can be formed directly from (1) olivine or orthopyroxene-dominated ultramafic rocks (i.e., Ol + 2CO_2aq_ = 2 Mgs + SiO_2_)^66,67^ or by (2) replacing antigorite (2Atg + 3CO_2_ = 3Mgs + Tlc + 3 H_2_O)^23,68^. We favour the second mechanism for our study because of a complete absence of quartz in the studied samples. This interpretation is supported by (1) the presence of fractures and vienlets of magnesite that crosscut serpentinites and antigorite groundmass (Supplementary Fig. S2c,d), which represent CO_2_-rich fluids pathways, (2) the presence of antigorite relics inside magnesite clasts (Fig. 1d), (3) the presence of minor talc associated with magnesite (Supplementary Fig. S2d), (4) strong similarities between the trace element patterns of magnesite and antigorite (Fig. 6), and (5) high trace element contents of magnesite over antigorite that support the formation of magnesite at higher temperature and depth (∼ 60–70 km)^68,69^ compared to the formation condition of antigorite. The carbon may have come from metamorphic decarbonation of subducted sediments^13,70^ as supported by similar FME and LREE patterns between the magnesite and subducted sediments, AOC and marine sediments. The presence of dolomite with magnesite indicates percolation of moderate to high flow of CO_2_-Mg-rich and Ca-poor fluids from the subducted sediments^66,67^. Generally, Mg-rich and Ca-poor fluids are associated with peridotites when they undergo complete or near-complete serpentinization^67^. Therefore, we suggest that magnesite formed after antigorites at higher temperatures and depths during subduction.

Although antigorite is the major carrier of trace elements in serpentinite, we note that magnesite has higher contents of FME of B, Li, As, Sb, Pb, Mo, Cs and LREE than antigorite and primitive mantle (Figs. 3, 6), which suggests that magnesite is a potential carrier of, as well as, a reservoir for these elements. In addition, magnesite is also a sink for Mn (Fig. 3). On the other hand, magnesite is depleted in Sr, Ba and U. In summary, we suggest that magnesite has high FME and LREE absorbing capacity of over 50–60% higher than serpentine phases (calculated according to differences in the contents of those elements between magnesite and antigorite). Based on our petrographic observations and previous experimental studies^68^, we argue that magnesite forms as a result of antigorite transformation, where the parent antigorite contributes a considerable amount of FME and LREE to the newly formed magnesite.

### Implications for arc magmatism and subduction polarity geochemical fingerprinting

**Figure 7:**
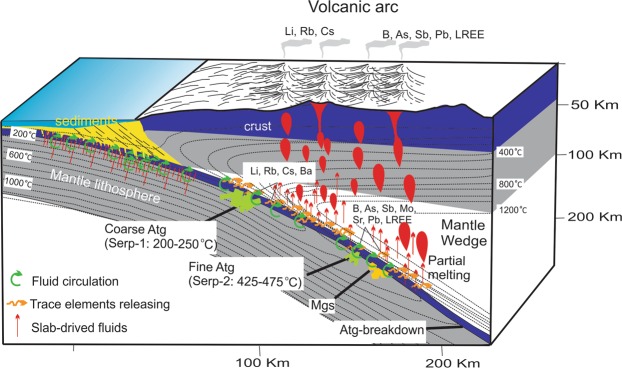
A cross-section sketch of a subduction zone complex showing the position of the first serpentinization stage (Serp-1) and formation of coarse antigorites (Atg) at 200–250 °C, the second serpentinization stage (Serp-2) and formation of fine antigorites at 425–475 °C and magnesite formation (Mgs).

The thermodynamic modelling results (Figs. 4, 5) demonstrate that the formation of two types of antigorite is a temperature-dependent process. The first serpentinization stage and formation of coarse antigorite is estimated at 200–250 °C and the second serpentinization stage and formation of fine antigorite occurred at 425–475 °C (Fig. 7). According to different FME and LREE contents of the two types of antigorite groups, we suggest that these elements mainly redistributed (uptaken, trapped and released) as a result of varying temperature. During the first serpentinization stage, the LILE such as Rb, Ba, Cs and Li and U are released from the subducted slab at low temperatures (200–250 °C) and shallow depths and incorporated into coarse antigorites (CA). These result are consistent with previous reported high enrichment of LILE in fluids released from subducting slabs at shallow depths and lower temperatures (~200 °C) directly beneath the forearc region^48,71^. The second serpentinization stage is represented by the release of higher amounts of B, As, Sb, Mo, Pb, Sr and LREE from the subducting slab at higher temperatures (425–475 °C) and greater depths, and their incorporation into the fine antigorites (FA) (Fig. 7).

Finally, we suggest that serpentinites remain stable at high sub-arc depths and represent a potential carrier of FME such as B, As, Sb, Sr, Mo, Pb and LREE that get recycled back into the mantle wedge through the so-called “antigorite breakdown” (600–700 °C)^4^. The similarities of the FME (B, As, Sb, and Li) patterns between the studied serpentinites (including rock forming minerals) and volcanic arc basalts (Fig. 6c) demonstrate how dehydration of serpentinites during subduction plays a principle role in the generation of arc-related magmatism (Fig. 7), in addition to the commonly considered dehydration of subducted sediments and AOC^48,62^. The high enrichment of these elements in the arc-related basalts can therefore be used to distinguish them from non-arc basalts such as plume-related basalt and MORB. The model also predicts that arc magmatism closest to the trench should have higher LILE such as Li, Rb and Cs, released during the first serpentinization stage, whereas those landward from the trench should be more enriched in B, As, Sb, Pb and LREE, released during the second serpentinization stage and after antigorite breakdown^4^. Such a cross-arc geochemical variation pattern has indeed been reported in some previous studies of arc volcanic rocks^72,73^. This cross-arc FME and LREE variation pattern, along with previously reported K_2_O/SiO_2_ cross-arc variation pattern^74^, can be powerful tools for the identification of subduction polarities of ancient arc systems.

## Methods

### Raman spectroscopy analysis

Raman spectra of serpentines and carbonates (Fig. 1e) were obtained by a micro-Raman system (HORIBA Jobin Yvon, Lab-RAM HR-800) equipped with a 514 nm Ar^+^ laser (melles Griot, 43 series Ion Laser, 543-GS-A02) and an optical microscope (Olympus, BX41) at Kanazawa university, Japan. The Ar^+^ laser has an irradiation power at 50 mW with a spectral resolution of about ± 2.5 to ± 3.5 cm^−1^. The Raman signal was acquired between 50 and 120 sec. Scattered light was collected in a backscattered geometry, using a pinhole (300 µm), slit (100 µm) and a grating (600 groves/ µm). A Si-based CCD (charge-coupled device) was used to record the Raman spectra. Based on OH stretching mode regions and lattice vibrational modes of the serpentine species^75^, the spectral regions (0 to 1500 cm^−1^ and 3400 to 3900 cm^−1^) were investigated. The LabSpec software was used to determine the band position of each spectrum.

### Bulk rock chemical analysis

Major and trace element bulk-rock geochemistry was carried out for 8 serpentinite samples (Supplementary Table; all whole-rock data is reported on a volatile-free basis). Whole-rock samples were crushed with a polyethylene-wrapped hammer into <0.5 cm pieces and then were ground with ethyl alcohol in an agate mill to a grain size below 50 μm. Major element compositions were analysed by X-ray fluorescence spectrometer (Shimadzu, XRF-1800) at the Pukyong National University, South Korea. Analytical conditions were 40 kV accelerating voltage and 70 mA beam current. Analytical precision is better than 2% for major elements. All glass beads were analysed three times and the averages were taken. Certified Reference Materials (CRMs), BIR-1 and JG-2, were used to determine the accuracy of the major element compositions. For detailed method for trace and rare earth element analyses see ref.^21^. Analytical values for USGS reference samples (BIR-1 and MUH-1) agree with recommended values within suggested tolerances. The precision of the measurements by repeated analyses of reference samples is better than ±5% for trace elements.

### Electron probe micro analysis (EMPA)

Major and some minor elements in antigorites and carbonates were acquired using a JEOL JXA-8800 electron-probe at Kanazawa University, Japan (Supplementary Table). The analytical conditions were 15 kV accelerating voltage, 20 nA probe current and 3 μm beam diameter. The ZAF-correction was performed to correct the raw data. Ferric and ferrous iron redistribution from electron microprobe analyses was made using the charge balance equation^76^. Natural minerals standards were used for calibration (JEOL Kanazawa STD1) such as Q17-quartz for Si, O16-corundum for Al, O19-eskolaite for Cr, M6-fayalite for Fe, O15-periclase for Mg, O20-manganosite for Mn, M8-wollastonite for Ca, M3-jadeite for Na, M13-KTiPO5 for K, Ti and O24-pentlandite for Ni. Olivine, clinopyroxene and spinel standard reference minerals, separated from Kurose peridotites xenoliths, were used.

### Laser ablation-inductively coupled plasma-mass spectrometry (LA-ICP-MS) micro analysis

Trace element compositions of serpentine and magnesite (Supplementary Table) were determined by 193 nm ArF Excimer LA-ICP-MS at Korean Basic Science Institute (KBSI), South Korea (Teledyne Cetac Technologies equipped with Analyte Excite). Analyses were performed by ablating 110 μm diameter spots at 10 Hz with an energy density of 5 J/cm^2^ per pulse. Signal integration times were 60 s for a gas background interval and 60 s for an ablation interval. The NIST SRM 612 glass was used as the primary calibration standard and was analysed at the beginning of each batch of <5 unknowns, with a linear drift correction applied between each calibration. The element concentration of NIST SRM 612 for the calibration is selected from the preferred values of Pearce *et al*.^77^. Each analysis was normalized using ^29^Si for serpentine and ^24^Mg for magnesite as internal standard elements, based on Si and Mg contents obtained by Electron probe micro analysis (EMPA). All minerals were analysed multiple times and the average taken. The relative standard deviations (RSD) of the trace and rare earth elements in the minerals were mostly 5–10%. To improve precision and accuracy of the trace elements data, we analysed the NIST612 CRM before and after each 5 unknown samples. High RSD is found only for ^137^Ba (10.7%).We analysed some clear homogenous areas of antigorite aggregates, but some area may contain dusty magnetite or other minor phases which may influence the actual antigorite trace elements values. Thus, we used the Glitter software, which shows ablation profile for element and permits to reduction of analytical contamination.

### Thermodynamic modelling

Thermodynamic modelling was calculated in a FMCASH-CO_2_ system (i.e., FeO-MgO-CaO-Al_2_O_3_-SiO_2_-H_2_O-CO_2_). Different pseudosections were constructed using Perple_X^78^ and the internally consistent dataset of Holland and Powell^79^. We assumed that local equilibrium, when chemical potentials are equalized^80^, was obtained at a length scale of millimetres. The solution model of (antigorite, chlorite, olivine and garnet, orthopyroxene, clinopyroxene, spinel, carbonate (magnesite) and fluid)^32,79,81,82^ was used. Ideal mixing is assumed for anthophyllite, brucite and talc. Lizardite and chrysotile were used without solution models. Equilibrium thermodynamics is widely used to study the tectonic evolution of the metamorphic rocks^79^. However, for serpentinite where mineralogical change is driven primary by fluid infiltration processes and to a lesser extent by changes in pressure and temperature, the thermodynamic techniques have been applied less commonly. Fluid infiltration process, in general, causes changes in the bulk rock composition, therefore the changes in the bulk chemistry of the system are considered in all the calculated pseudosections. All the major elements of the modelled chemical system show linear relations with the H_2_O content (Supplementary Fig. S3). Al_2_O_3_, SiO_2_ and FeO show negative correlations while MgO shows a positive correlation. The concentrations of these elements were shifted from the original composition of the rock during partial melting and serpentinization process^21^. For the modelling, the x-axes of the pseudosections are assigned to the composition of the system. The value zero of the x-axes indicates the less modified amounts of the oxides while the value one indicates the maximum modification recorded in the studied rock samples.

## Supplementary information

SUPPLEMENTARY text

SUPPLEMENTARY Table
