# Supplementary material for: Origin of arc magmatic signature: A temperature-dependent process for trace element (re)-mobilization in subduction zones
Source: Sci Rep. 2019 May 8;9:7098. doi: 10.1038/s41598-019-43605-9 (PMC6506526; doi:10.1038/s41598-019-43605-9)
Supplement: Supplementary file 1 — SUPPLEMENTARY text [file 41598_2019_43605_MOESM1_ESM.pdf]

Supplementary information for:

Origin of arc magmatic signature: A temperature-dependent process for trace element (re)-mobilization in subduction zones

Hamed Gamal El Dien[1,2,*], Zheng-Xiang Li[1], Yongwoo Kil[3], Tamer Abu-Alam[4]

[1] Earth Dynamics Research Group, The Institute for Geoscience Research (TIGeR) and ARC Centre of Excellence for Core to Crust Fluid Systems (CCFS), School of Earth and Planetary Sciences, Curtin University, GPO Box U1987, Perth, WA 6845, Australia

[2] Geology Department, Faculty of Science, Tanta University, 31527 Tanta, Egypt

[3] Department of Energy and Resources Engineering, College of Engineering, Chonnam National University, Yongbong-ro, Buk-gu, Gwangju, South Korea

[4] Universitetsbiblioteket, University of Tromsø - The Arctic University of Norway, 9037 Tromsø, Norway

[*] Corresponding author:

E-mail address: hamed.gamaleldien@postgrad.curtin.edu.au

Tel: +61405853493

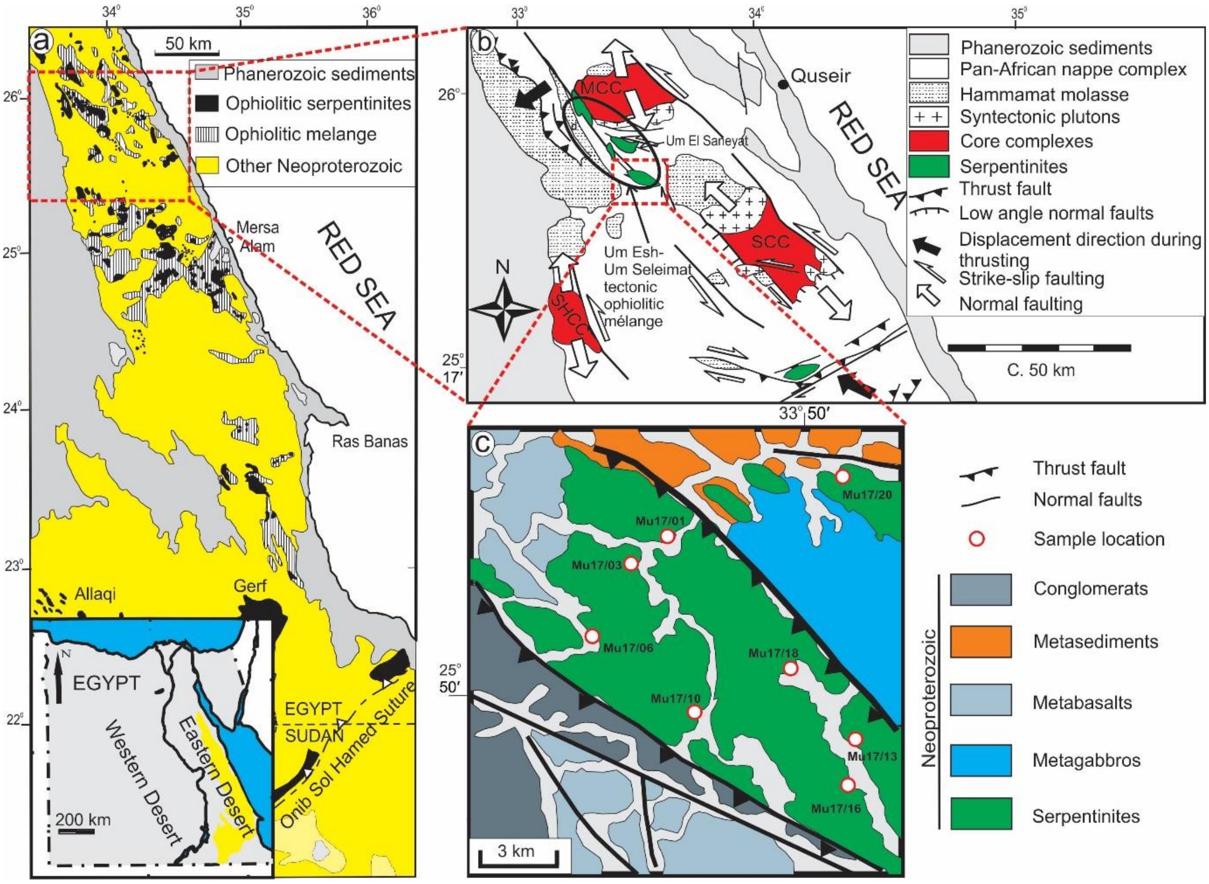

Figure S1: (a) Distribution of the ophiolites in the Arabian-Nubian Shield (modified after Vail[1]). (b) Location of the studied serpentinite masses and their relations, with the major structural trends and younger metamorphic and magmatic core complexes (MCC, Meatiq and SCC, Sibai) in the East-African Orogen, central Eastern Desert of Egypt (major structures are after Fritz et al.,[2] and Abd El-Wahed and Kamh[3]). (c) Geologic map of the Muweilih serpentinites in the Wadi Muweilih area (modified after El Bahariya[4]) .

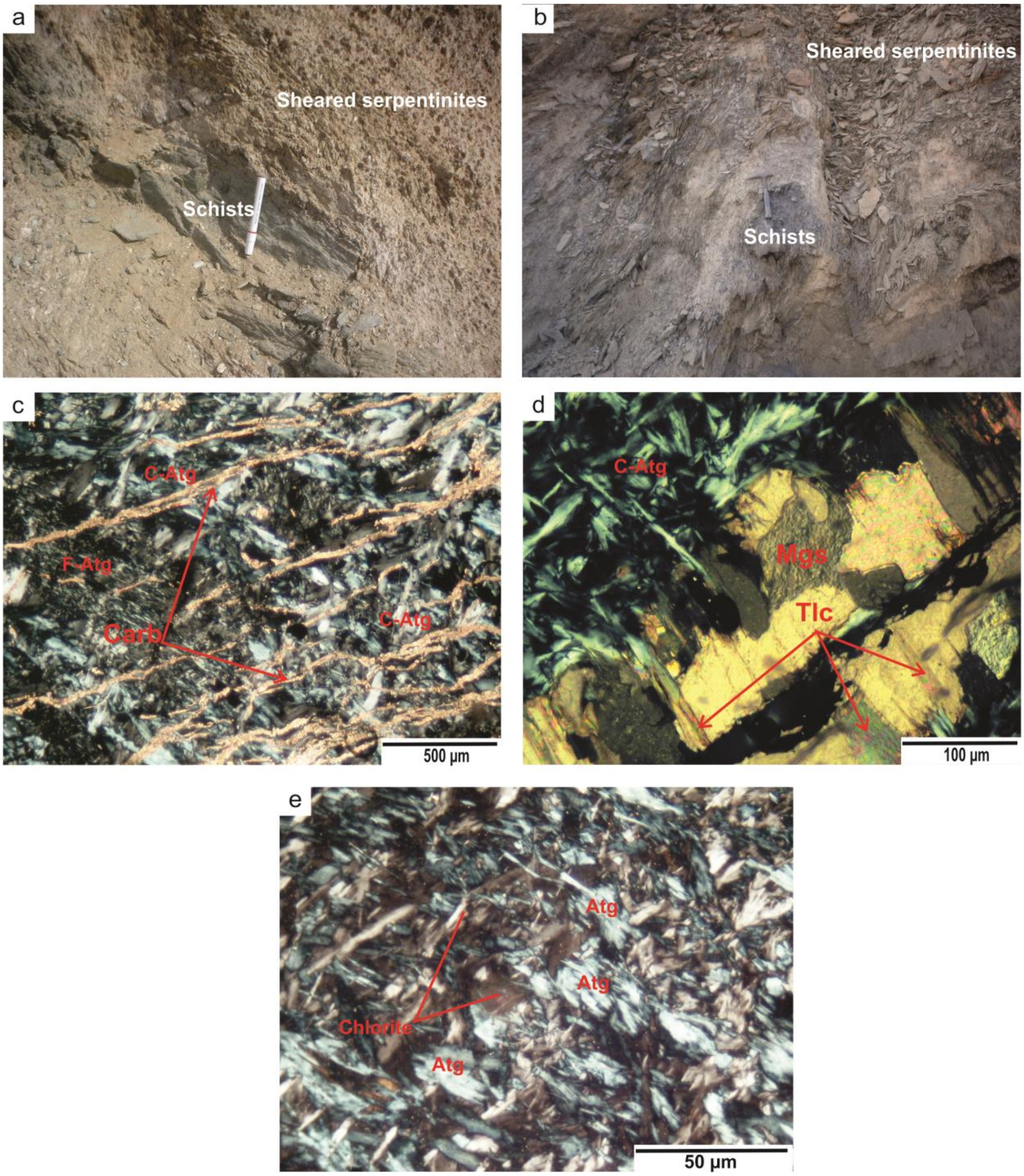

Figure S2: (a, b) Field photos showing a tectonic melange consisting of highly sheared serpentinites and schists. Photomicrographs of the studied serpentinites. (c) Carbonates (Carb) veins crosscut the antigorite (F-Atg and C-Atg) groundmass, indicating a later formation age after antigorite. (d) Magnesite (Mgs) veins with small amounts of talc (Tlc). (e) Chlorite aggregates disseminated in the antigorite groundmass.

Bulk-rock composition

All the examined rocks are completely serpentinized with high-water contents (i.e., high LOI values of 12.54 to 14.93 wt%)(Fig. S3). The MgO contents of the studied samples show a range of 40.47 wt % (sample # MU17/06) to 45.67 wt% (sample # MU17/16) (Fig. S3). Al_2O_3 and SiO_2 contents have ranges of 0.64–1.81 wt. % and 44.85–46.84 wt%, respectively. Fe_2O_3 and MnO contents are in the ranges of 8.49–10.20 wt% and 0.04–0.35 wt%, respectively (Supplementary Table S1). Trace elements spider-diagram, normalized to Primitive Mantle

(PM) of McDonough and Sun[5], displays low contents of highly and moderately compatible elements relatively to the high incompatible elements (Fig. S4). Trace elements concentrations vary among the samples. All the studied serpentinite rocks show decreasing slope from the fluid mobile elements (FME) to rare earth elements (REE) (Fig. S4). As, Ba, Cs, U, Pb, Sr, Li, Mo and Hf show positive spikes relative to elements of the same compatibility. Chondrite (CI)-normalized REE patterns in all the studied serpentinites, using the normalizing values of Anders and Grevesse[6], display nearly-flat shapes (Fig. S4). Light rare earth elements (LREE) are enriched in comparison to middle rare earth elements (MREE), and there is a slight enrichment form Sm to La (La_N/Sm_N = 1.92–3.91; La_N/Yb_N = 0.17–4.50 CI-Chondrite). Heavy rare earth elements (HREE) show a slight negative slope to MREE. The HREE concentrations reflect the fertility of the samples ($Yb_{N(\text{CI-normalized value})}$ = 0.01–0.31).

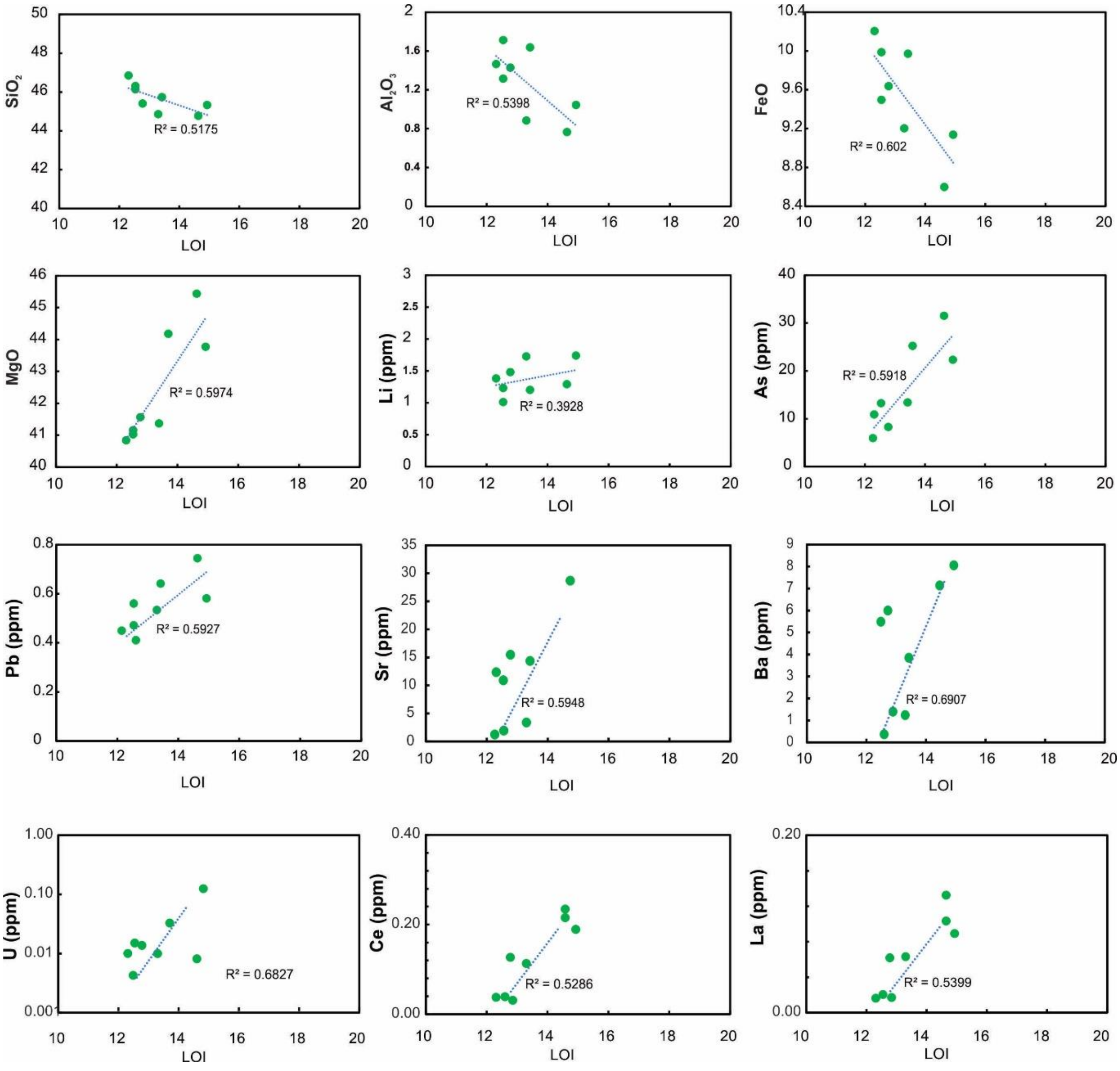

Figure S3. Variation diagrams of LOI (wt%) vs. selected major, trace and rare earth elements of bulk composition of the serpentinites.

Nature of protolith

To trace the primary mantle processes in ultramafic rocks that underwent intense serpentinization and subduction metamorphism, we checked the whole-rock trace (i.e., HREE) element compositions of the samples because they are fluid immobile HREE of the whole-rock

composition, particular Yb, are highly immobile during post-melting processes and fluid circulation in mantle peridotite[7–9]. Their contents are commonly used to estimate melting degrees of the original fertile source[10]. The co-variations between Yb and some major and trace elements such as Al, Y, Sc, V, Cr and Zr are almost the same during partial melting and have lower contents than the primitive mantle[5] and the depleted MORB mantle(DMM)[11] (Fig. S5). This suggests a residual origin of mantle protolith of the studied serpentinites. The correlations between Yb and Cr, Sc and V (Fig. S5c, d, e) demonstrate that the mantle protolith of the studied serpentinites have high melting degrees ranging between ~16% and 27 %. Such estimated melting degrees agree with our results of non-modal fractional melting modelling in the spinel stability field[11] using the HREE of the studied samples and the DMM (Fig. S4) and unaltered spinel core Cr-numbers. Generally, V values record the fO_2 during mantle melting[12]. The V values from the studied serpentinite rocks plot around the QFM+1 curve indicating oxidizing conditions (Fig. S5e).

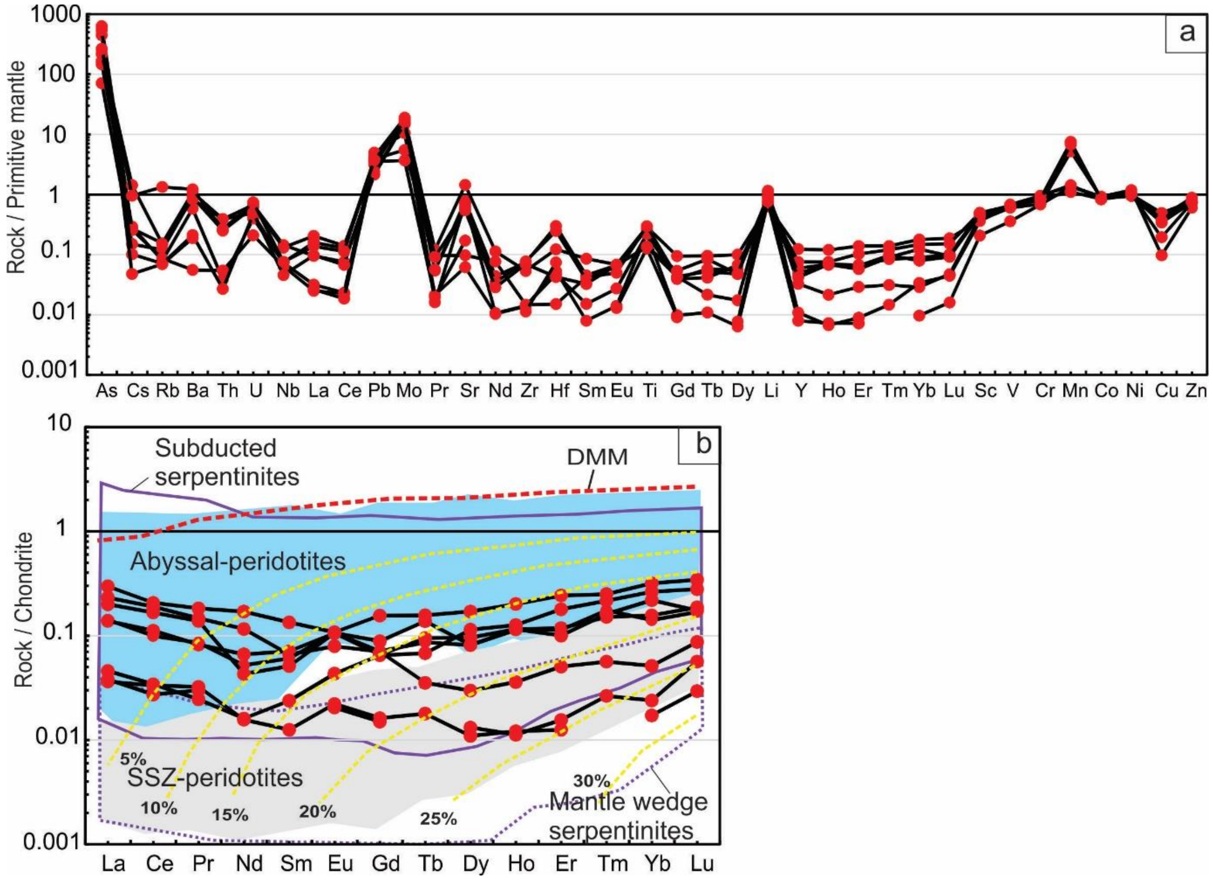

Figure S4: Primitive mantle[5] normalized multielement and rare earth element patterns normalized to chondrite[6] of the studied serpentinites. Fields of Abyssal peridotites are from Niu[9] , supra subduction zone (SSZ; Izu-Bonin-Mariana) peridotites from Parkinson and Pearce[10] and Subducted and mantle wedge serpentinites from Deschamps et al.[13]. The non-modal fractional melting model of a depleted MORB mantle (DMM)source[11] is used, with melting curves assumed of a of DMM source in the spinel stability field[8].

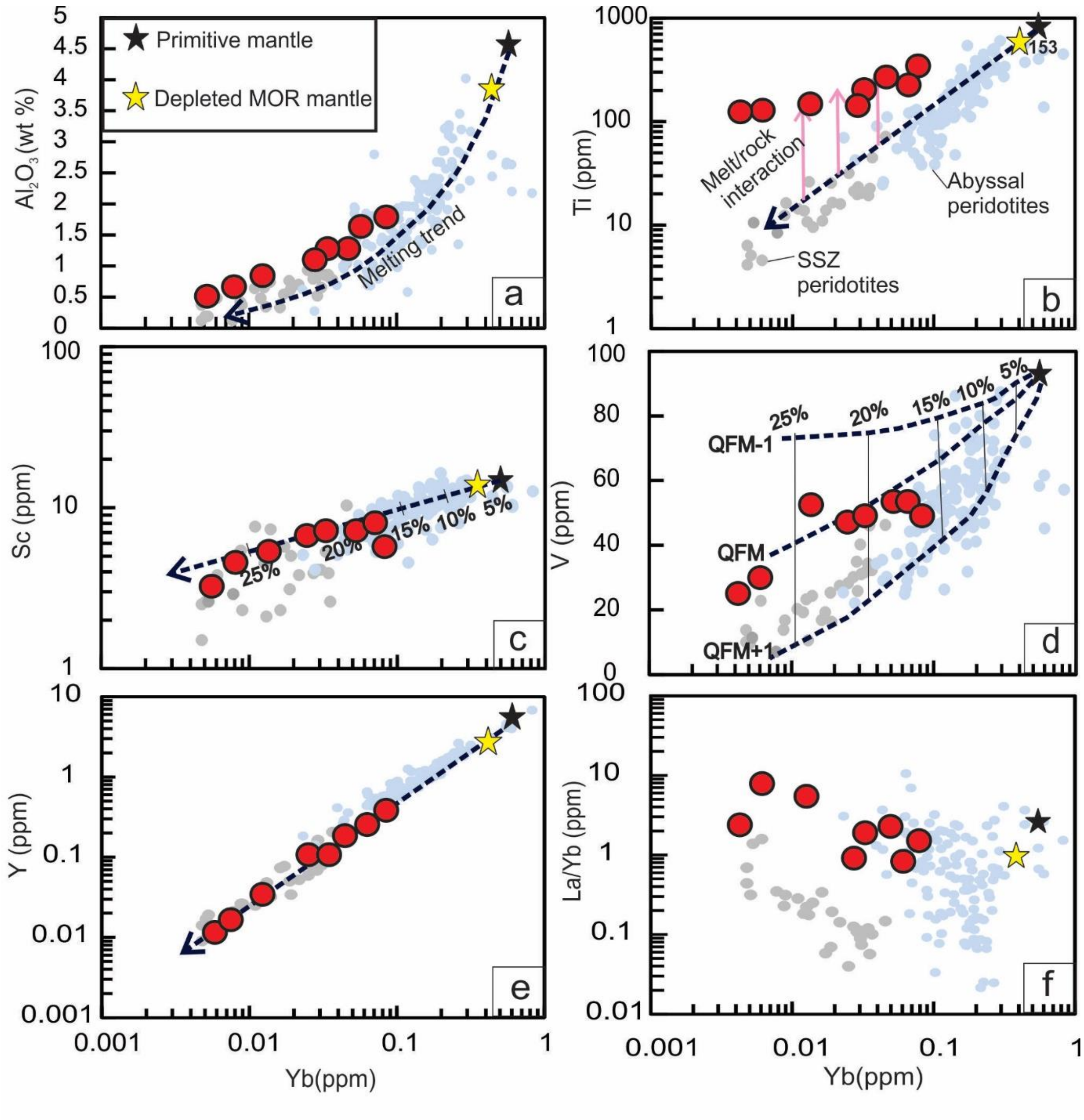

Figure S5: Whole-rock Yb (ppm) vs. selected major, trace and rare earth elements variation of the studied serpentinites. Fields of abyssal peridotites are from Niu[9], and supra subduction zone (SSZ; Izu-Bonin-Mariana) peridotites from Parkinson and Pearce[10]. Compositions of the primitive mantle[5] and depleted MORB mantle (DMM)[11] is plotted for comparison.. Determination of the degree of partial melting was based on bulk-rock chemistry showing in figures (c, d) according to the bivariates of Yb (ppm) vs. Cr and V (ppm) for residual peridotites, according to Lee et al.[12], with annotated degrees of mantle melting (in %). Fractional melting trends for different oxygen fugacities are also shown. FMM = fertile MORB mantle. Vanadium behaves as a moderately incompatible element when melting occurs under reducing conditions (QFM–1; this refers to log fO_2 (QFM) = log units relative to quartz–fayalite–magnetite buffer), resulting in low depletion of V. Under oxidizing conditions (QFM+1), the ratio of $V^{3+}/(V^{4+}+V^{5+})$ become lower whereas partition coefficients are higher causing vanadium to become a highly incompatible element and readily depleted in the mantle during partial melting. The melt/rock interaction trend in (b) after Deschamps et al.[13] Depletion trends are after Uysal et al.[14] and Gamal El Dien et al.[15]

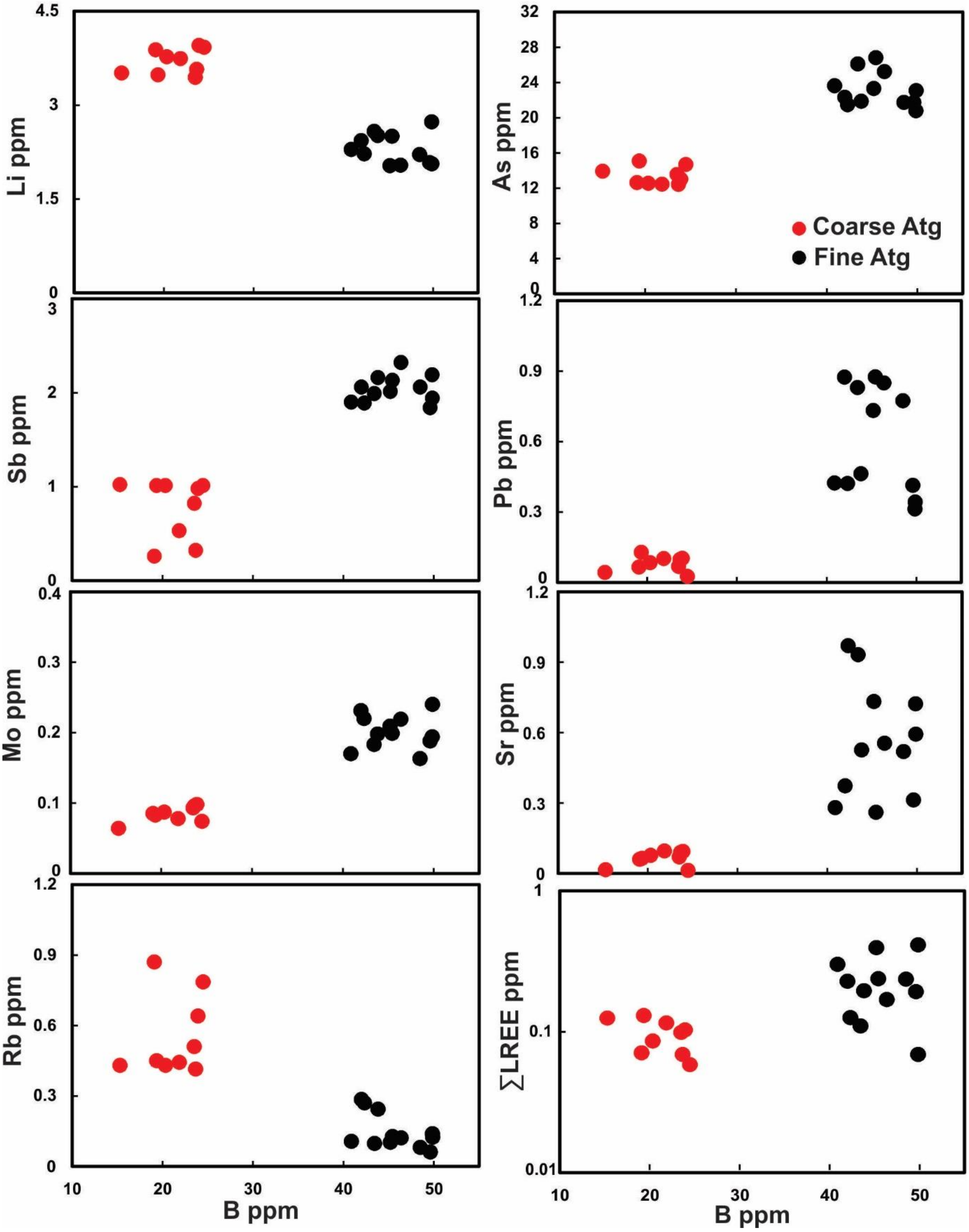

Figure S6: Binary plots of B and various trace elements in the studied antigorites showing the different contents between the two antigorites groups. All concentrations are in ppm.

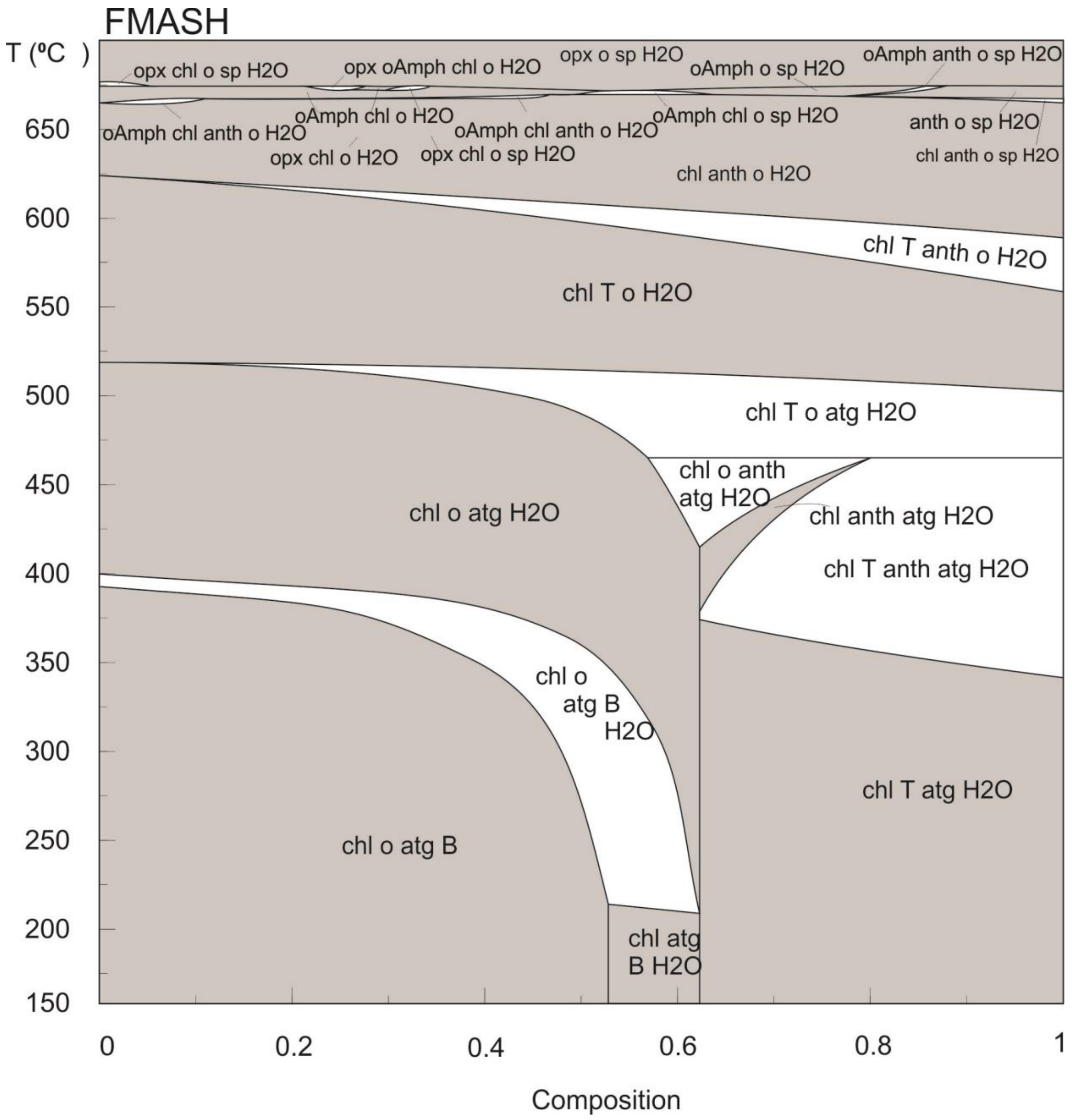

Figure S7: A T-X pseudosection at 2 kbar shows the stability range of the spinel mineral up to > 670 ºC. This indicates that the observed spinel in the studied samples are a metastable phase with respect to the surrounding low-grade assemblage. The x-axe is the bulk composition which is similar to that in Figures. 4 and 5 in the main text with the exception of reducing the system to FMASH.

References

1. Vail, J. R. Pan-African crustal accretion in north-east Africa. *J. African Earth Sci.* **1,** 285–294 (1983).

2. Fritz, H. *et al.* Orogen styles in the East African Orogen: A review of the Neoproterozoic to Cambrian tectonic evolution. *J. African Earth Sci.* **86,** 65–106 (2013).

3. Abd El-Wahed, M. A. & Kamh, S. Z. Pan-African dextral transpressive duplex and flower structure in the Central Eastern Desert of Egypt. *Gondwana Res.* **18,** 315–336 (2010).

4. El Bahariya, G. A. Classification and origin of the Neoproterozoic ophiolitic mélanges in the Central Eastern Desert of Egypt. *Tectonophysics* **568–569,** 357–370 (2012).

5. McDonough, W. . & Sun, S. –. The composition of the Earth. *Chem. Geol.* **120,** 223–252 (1995).

6. Anders, E. & Grevesse, N. Abundances of the elements: Meteoritic and solar. *Geochim. Cosmochim. Acta* **53,** 197–214 (1989).

7. Pearce, J. A., Barker, P. F., Edwards, S. J., Parkinson, I. J. & Leat, P. T. Geochemistry and tectonic significance of peridotites from the South Sandwich arc-basin system, South Atlantic. *Contrib. to Mineral. Petrol.* **139,** 36–53 (2000).

8. De Hoog, J. C. M., Janák, M., Vrabec, M. & Froitzheim, N. Serpentinised peridotites from an ultrahigh-pressure terrane in the Pohorje Mts. (Eastern Alps, Slovenia): Geochemical constraints on petrogenesis and tectonic setting. *Lithos* **109,** 209–222 (2009).

9. Niu, Y. Bulk-rock major and trace element compositions of abyssal peridotites: Implications for mantle melting, melt extraction and post-melting processes beneath Mid-Ocean ridges. *J. Petrol.* **45,** 2423–2458 (2004).

10. Parkinson, I. J. & Pearce, J. A. Peridotites from the Izu – Bonin – Mariana Forearc (ODP Leg 125): Evidence for Mantle Melting and Melt – Mantle Interaction in a Supra-Subduction Zone Setting. *J. Petrol.* **39,** 1577–1618 (1998).

11. Workman, R. K. & Hart, S. R. Major and trace element composition of the depleted MORB mantle (DMM). *Earth Planet. Sci. Lett.* **231,** 53–72 (2005).

12. Lee, C.-T. A., Brandon, A. D. & Norman, M. Vanadium in peridotites as a proxy for paleo-fO2 during partial melting: prospects, limitations, and implications. *Geochim. Cosmochim. Acta* **67,** 3045–3064 (2003).

13. Deschamps, F., Godard, M., Guillot, S. & Hattori, K. Geochemistry of subduction zone serpentinites: A review. *Lithos* **178,** 96–127 (2013).

14. Uysal, I., Ersoy, E. Y., Dilek, Y., Kapsiotis, A. & Sarifakioğlu, E. Multiple episodes of partial melting, depletion, metasomatism and enrichment processes recorded in the heterogeneous upper mantle sequence of the Neotethyan Eldivan ophiolite, Turkey. *Lithos* **246–247,** 228–245 (2016).

15. Gamal El Dien, H. *et al.* Neoproterozoic serpentinites from the Eastern Desert of Egypt: Insights into Neoproterozoic mantle geodynamics and processes beneath the Arabian-Nubian Shield. *Precambrian Res.* **286,** 213–233 (2016).
